# Rare specimen identification in an un-integrated taxonomy: implications of DNA sequences from a Taiwanese *Philine* (Mollusca, Philinidae)

**DOI:** 10.3897/zookeys.1060.28809

**Published:** 2021-09-17

**Authors:** Donald J. Colgan, Shane T. Ahyong, Karine Mardon, Ian M. Brereton

**Affiliations:** 1 Australian Museum Research Institute, 1 William St., Sydney 2010 Australia Australian Museum Research Institute Sydney Australia; 2 School of Biological, Earth & Environmental Sciences, University of New South Wales, Kensington, NSW 2052, Australia University of New South Wales Kensington Australia; 3 Centre for Advanced Imaging/National Imaging Facility, University of Queensland, Brisbane 4072, Australia University of Queensland Brisbane Australia

**Keywords:** Gizzard plates, 16S ribosomal RNA, micro-CT scanning, Scaphopoda

## Abstract

Many species of the gastropod genus *Philine* have been named from northeastern Asia but scanty descriptions based predominantly on shells make it difficult to determine which are valid. This, plus the sporadic anatomical and genetic information available for many of these species has led to what may be described as an un-integrated taxonomy. In this situation, it is generally preferable to postpone dissection of rare and unusual specimens until relevant diagnostic characters can be established in broader studies. Micro-CT scanning and DNA sequencing were used to examine such a specimen collected recently from deep waters off northeastern Taiwan. Micro-CT examination of the morphology of the internal shell and gizzard plates suggested that, among named species, the sequenced specimen is most similar to *P.otukai*. It cannot, however, be definitively referred to *P.otukai* as that species lacks adequate anatomical description or known DNA sequences. Phylogenetic analyses of newly collected DNA sequences show the specimen to be most closely related to, but distinct from the northern Atlantic Ocean and Mediterranean species, *Philinequadripartita*. The sequences also confirm genetically that five or more species of *Philine* occur in northeast Asia, including at least three subject to considerable taxonomic uncertainty.

## Introduction

Accurate delimitation of species of the highly speciose cephalaspidean genus *Philine* Ascanius, 1772 is hampered by the brief original descriptions of many of its named taxa. Many such descriptions were made using only the shell, which has limited diagnostic value, and often these have not been subsequently supplemented by studies of internal anatomy, including morphology of the diagnostically important gizzard plates, or DNA sequences.

The difficulty of delimitation is especially marked in northeastern Asia, as exemplified by the situation in Taiwan from which three named *Philine* species (*P.argentata* Gould, 1859; *P.vitrea* Gould, 1859, and *P.otukai* Habe, 1946) are currently recognised (Lee 1998; Wu 2004; Wu and Lee 2005). We have recently collected a specimen from deep waters off Taiwan that is not readily identifiable owing to the lack of an adequate taxonomic description of comparable species. A fourth species, *Philinekurodai* Habe, 1946, has been recorded in Taiwan but the relevant specimens were supposed to belong to *P.otukai* according to Lee (1998) and Wu (2004). Many other species of *Philine* have been named from nearby regions, and in total, 15 named recent species with medium to large shells have been recorded (Table 1) from the coasts of China, Korea, and Japan (Zhang 2008; Price et al. 2011; Chaban 2014; Chaban and Chernyshev 2014; Chaban et al. 2019; and references in these articles). The anatomical and genetic information available for most of these species is sporadic, resulting in a taxonomy that may be described as un-integrated, with a general lack of diagnostic characters suitable for species discrimination. In this situation, we consider it advisable to minimise destructive sampling of rare specimens until such diagnostic characters can be established from more frequently collected taxa.

**Table 1.** *Philine* species from northeastern Asia with medium- or large-sized shells. Localities of identified type specimens are followed by (T). Information categories are abbreviated as: D, DNA sequence; G, gizzard plate morphology, O, other anatomical.

Species	Locality	Information	Status
*P.acutangula* Adams, 1862	Gulf of Lian-Tung, Hulu-Shan Bay, China [Gulf of Liadong, Hulushan Bay]	No figure or type description	Species inquirendum
*P.argentata* Gould, 1859	Hokkaido Bay, Japan (T)	G, O: Chaban (2014)	Synonym of *P.orientalis* (Price et al. 2011); species status maintained by Chaban (2014)
		D: Chaban et al. (2019)	
*P.coreanica* Adams, 1855	Coreen archipelago on mud flats [Korea]	Sowerby (1855) fig. 166 (figured type?)	Species inquirendum: “*P.aperta*” fide Herdman (1906)
*P.crenata* Adams, 1862	Tsu-Shima, Korea Strait, 46 m		Species inquirendum: “*P.aperta*” fide Herdman (1906)
*P.habei* Valdés, 2008	Fiji, 17°05'S, 178°55'W, 654–656 m (T)	D: Oskars et al. (2015)	Accepted
		G, O: Valdés (2008)	
*P.japonica* Lischke, 1872	Jedo (Tokyo Bay), Japan (T)	D: Chaban et al. (2019)	*P.orientalis* synonym (Price et al. 2011). Species status maintained by Chaban et al. (2019)
		O: Larvae: Hamatani (1961) and Tanaka (1958).	
*P.kinglipini* Tchang, 1934	Bays of Tsangkou and Nukukou	G, O: Tchang (1934)	Accepted. No type designated.
*P.kurodai* Habe, 1946	Wakayama Prefecture, Japan (T)		Accepted by Chaban (2016)
*P.miyadii* Habe, 1946			Synonym of *P.scalpta* fide Habe (1950) (= *P.vitrea* (see below)
*P.orientalis* Adams, 1854	“Eastern seas” (T)	G, O: Price et al. (2011)	Accepted
		D: Krug et al. (2012)	
*P.otukai* Habe, 1946	Penghu Islands, Taiwan (T)		Accepted
*P.paucipapillata* Price, Gosliner & Valdés, 2011	Kampote and Prek Romeas, Cambodia (T)	G, O: Price et al. (2011)	
		D: Krug et al. (2012)	
*P.scalpta* A. Adams, 1862	Tsu-Shima and Korea Strait 50–80 m	D: Chaban et al. (2019)	Synonym of *P.vitrea* (Higo et al. 1999). Species status maintained by Chaban and Chernyshev (2014).
*P.striatella* Tapparone-Canefri,1874	Yokohama, Japan (T)	O (radula): Tapparone-Canefri (1874)	Synonym of *P.japonica* fide Pilsbry (1895), or *P.orientalis* fide Price et al. (2011)
*P.vitrea* Gould, 1859	Hong Kong (T)		Accepted

We used non-destructive morphological analysis of the collected specimen and DNA sequencing of a small external tissue sample to make initial comparisons with named species. For morphological analysis, we used micro-CT scanning (Shepelenko et al. 2015) which is potentially very useful for studying specimens where dissection would be premature. We focussed on the shape and sculpture of the shell, which is internal in *Philine*, and the three gizzard plates which have been applied to morphological diagnoses of the genus (e.g., Price et al. 2011).

We determined sequences of 16S ribosomal RNA (16S rRNA), histone H3 (H3), and the D1 expansion region of 28S ribosomal RNA (D1 28S rRNA). We then performed phylogenetic analyses of the sequences, including sequences from previous DNA studies of the Philinidae (Krug et al. 2012; Ohnheiser and Malaquias 2013; Gonzales and Gosliner 2014; Oskars et al. 2015; Chaban et al. 2019). We were unable to sequence the cytochrome *c* oxidase subunit I bar-coding region, a difficulty also found by Krug et al. (2012), possibly explaining why there are relatively few GenBank accessions for this gene from *Philine*.

## Materials and methods

### Material

AMS C.559479, off Dasi, Yilan County, north-east Taiwan, 24°54'N, 122°E, 5 xi 2015, coll. S. Ahyong, commercial bottom trawl, 300–400 m depth, fixed and stored in 80% ethanol. The specimen was collected together with other deep-water species including the crustaceans, *Bathynomusdoederleini* Ortmann, 1894, *Homolaorientalis* Henderson, 1888, and *Metanephropsthomsoni* (Bate, 1888).

### Measurements

Digital callipers were used for external measurements, made after preservation. Measurements of the relative sizes of internal structures, such as gizzard plates, were determined from images using the beta 4.0.3 version of Scion Image (Scion Corporation, Frederick, MD, USA). Sizes of internal structures were estimated by multiplying the ratio of the relative lengths of the relevant structure and the longest shell axis on an image by the length of this axis determined by callipers on the actual specimen.

### Micro-CT imaging

Micro-CT scanning was performed directly on the specimen after removal from ethanol storage with a Siemens Inveon micro-CT scanner operated at 80 KV energy, 250 µA intensity with 540 projections per 360° and 2200 ms exposure time. The sample was scanned at a nominal isotropic resolution of 27.8 µm. The data were reconstructed using a Feldkamp conebeam back-projection algorithm provided by an Inveon Acquisition Workstation from Siemens.

### DNA methods and molecular phylogenetic analysis

DNA was extracted from a small amount of mantle tissue using the Bioline Isolate II Kit following the manufacturer’s instructions. The final centrifugation was performed with 100 µL of the kit’s elution buffer. PCR amplifications followed the methods of Colgan and Da Costa (2009) using the following primers:

16S rRNA: 16Sar CGCCTGTTTATCAAAAACAT (Palumbi 1996), 16sbr CCGGTCTGAACTCAGATCACGT (Palumbi 1996)

28S rRNA: 28S D1F ACCCSCTGAAYTTAAGCAT (McArthur and Koop 1999) 28S D1R AACTCTCTCMTTCARAGTTC (Colgan et al. 2003)

H3: H3NF ATGGCTCGTACCAAGCAGAC (Colgan et al. 2000)

H3NR ATRTCCTTGGGCATGATTGTTAC (Colgan et al. 2000)

PCR products were checked by electrophoresis on 2% agarose gels run with TAE buffer, including Gel Red (Biotium Inc., Fremont, CA) and visualised with UV-fluorescence. Single-banded products of the appropriate size were purified by Exo-SAP-IT^TM^ (Thermo-Fisher Scientific) and sequenced commercially at Macrogen (Seoul, Korea) with the primers used in the original amplification.

GenBank Accession Numbers for new sequences: MH340050 (D1 28S rRNA), MH340051 (16S rRNA), MH340052 (histone H3).

Phylogenetic analyses were conducted using all *Philine* sequences for each gene available on 20 September 2020. Sequences from *Philinopsis* Pease, 1860 were used to root analyses for the 16S rRNA dataset. The sets of sequences for each gene were aligned with ClustalW (Thompson et al. 1997).

Maximum likelihood analyses (ML) analyses were conducted at the CIPRES Science Gateway (https://www.phylo.org/portal2/home.action, Miller et al. 2010) using the RAxML Blackbox (Stamatakis et al. 2008), assuming no invariable sites, using empirical base frequencies and assessing node support by rapid bootstrap with the number of replicates determined by the software, according to the MRE-based bootstopping criterion (Pattengale et al. 2010). Analyses of the complete 16S rRNA data were also run on a filtered alignment for which GBlocks (Castresana 2000) was used to remove less well supported sections, allowing gap positions within the final blocks and less strict flanking positions. Analyses were also conducted for a reduced set of 16S rRNA sequences comprising members of a strongly supported clade of taxa allied with *P.aperta*. MEGA 7.0 (Kumar et al. 2016) was used to conduct other analyses including calculation of genetic distances and minimum evolution phylogenetic trees.

## Results

### Morphology

**Figure 1.**
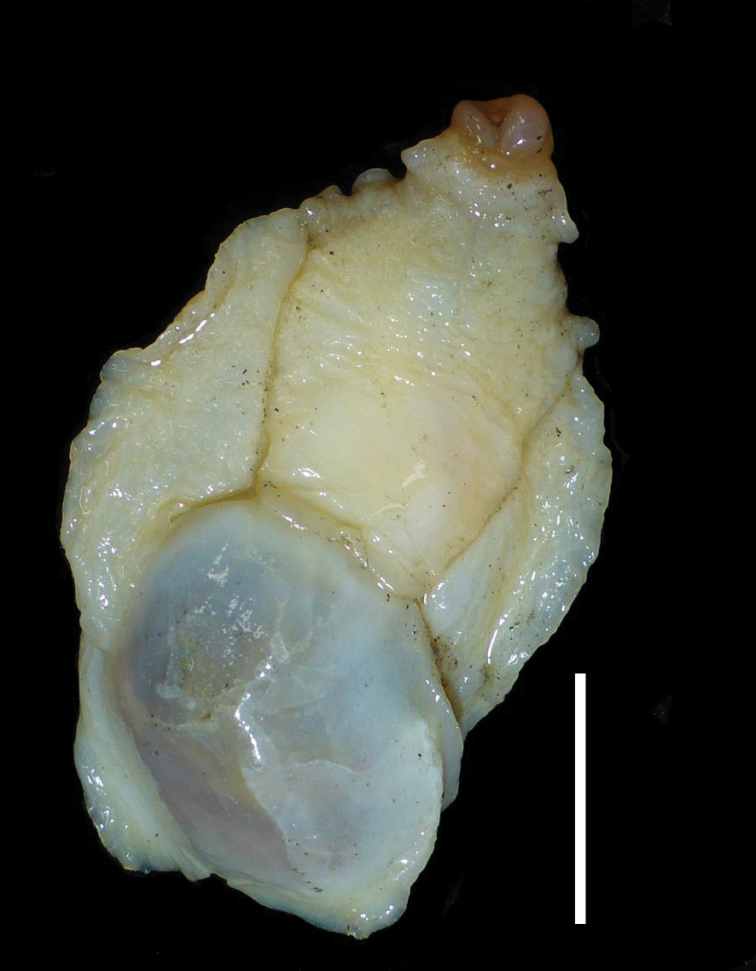
C.559479, freshly caught animal (photograph Shane Ahyong). Scale bar: 10 mm.

**Animal, external**: (Fig. 1) **Colour.** headshield creamy yellow, parapodia paler, foot off-white. Headshield: anterior three-fifths transversely rugose; central anterior to posterior furrow more marked posteriorly; dividing into two short processes posteriorly, overlapping posterior shield very slightly; tapering posteriorly in dorsal view (length 15.6 mm, width at widest 12.2 mm, width at shell 9.00 mm). Parapodial lobes broad, not remarkably thickened. Foot extending slightly posterior to shell. Posterior shield mostly occupied by a thinly covered internal shell. Posterior shield appearing grey-black due to pigmentation of internal organs, maintained after fixation. **Dimensions**: Animal 35.3 mm long (buccal mass everted) and 21.5 mm wide when newly collected (measured from photograph). After fixation: length from front of headshield to tail of foot 34 mm, width 19.8 mm and maximum height 9.3 mm.

**Figure 2.**
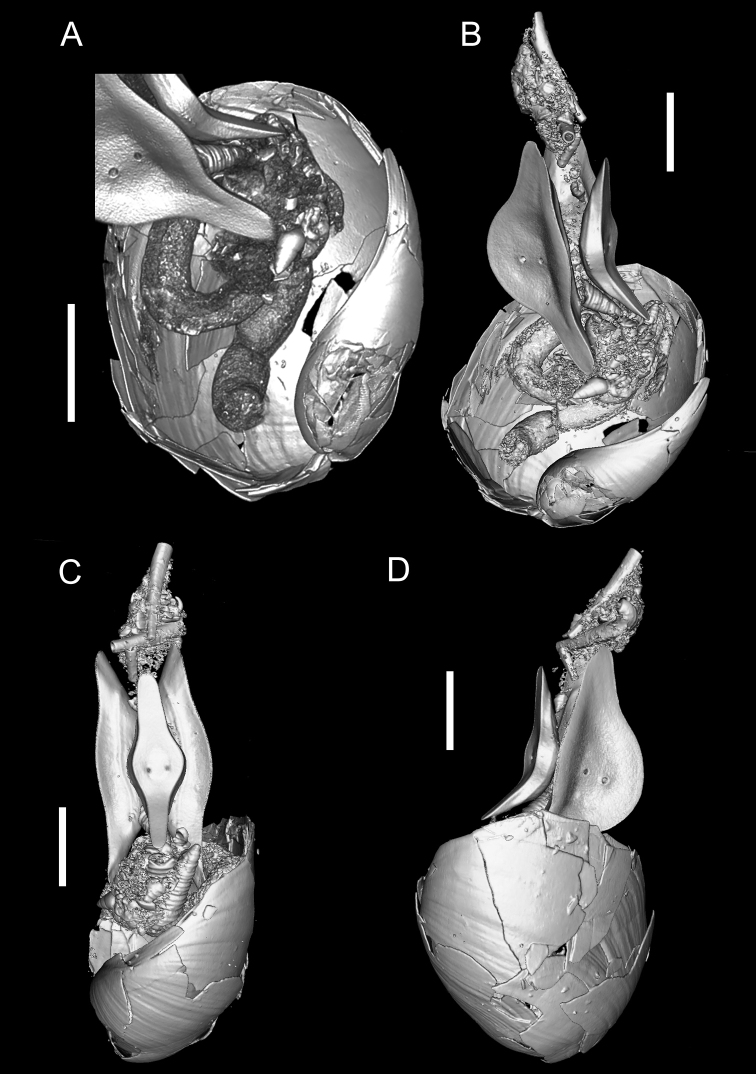
Micro-CT reconstruction images of C.559479 **A** ventral view of the shell **B–D** three perspectives from the reconstruction. Scale bar: 5 mm (**A–D**).

**Shell**: (Fig. 2) fragile, internal, height of body whorl, measured vertically from the posterior end of the aperture to the tip of the (damaged) elevated anterior outer lip: 18.4 mm, width of body whorl 14.2 mm, measured by callipers; spire involute, aperture extremely wide and rounded below, upper margin rising above shell vertex, columellar lip notably reflected, albeit slightly, (Fig. 2A). Frequent irregularly-spaced curved growth lines and spiral ribs, covering most of body whorl (except for bottom quarter). Radial and spiral ribs sometimes intersecting to form weak, irregularly cancellate sculpture (Suppl. material 2: Fig. S1).

**Figure 3.**
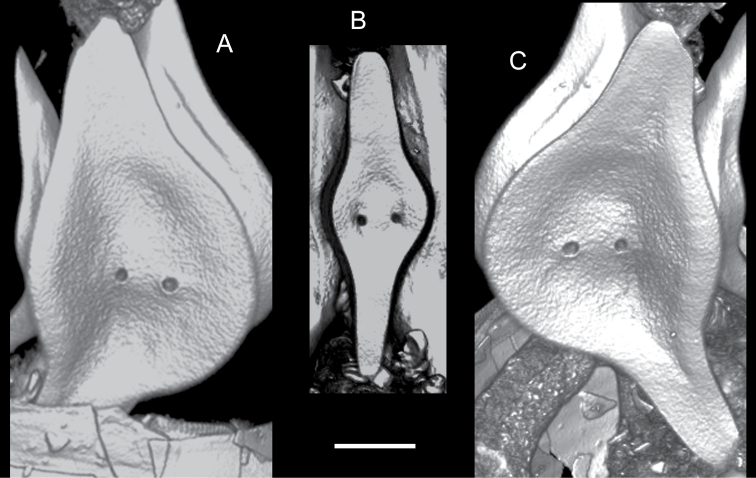
C.559479, outer surface of gizzard plates from the micro-CT reconstruction **A** paired plate, left **B** unpaired plate **C** paired plate, right. Scale bar: 2 mm (**A–C**).

**Gizzard plates**: (Figs 2B–D, 3). One pair of similar plates (12.5 mm in longest dimension) and notably smaller, unpaired plate (9.0 mm in longest dimension), all with outer surface (that facing towards the animal’s surface) slightly dimpled. Plates spindle-shaped, each with two large, circular pores in central area markedly depressed inwards. Smallest plate with spindle arms aligned axially; axes of spindle arms of larger plates form an obtuse angle (~ 135°). Spindle arms unequal in length (measured from the midpoint of the line between the two pores in each plate). Ratio of arm lengths: 1.15 for the small plate; 1.18 for paired plates. Posterior arm of all plates more slender than anterior arm.

### Gut contents

The prey of *Philine* are predominantly bivalves (Morton and Chiu 1990) but gastropods are also taken. For example, *P.orientalis* is known to prey on at least 11 species of small snails (Taylor 1982). The diet of C.559479 included multiple species of micro-gastropods (at least three are observable in Fig. 2B, including the sparsely pustulated micro-gastropod between the gizzard plates) and scaphopods (at the mouth in Fig. 2C and in the Suppl. material 1). We are not aware of previous reports of Philinidae feeding on this class of molluscs.

### DNA

**Figure 4.**
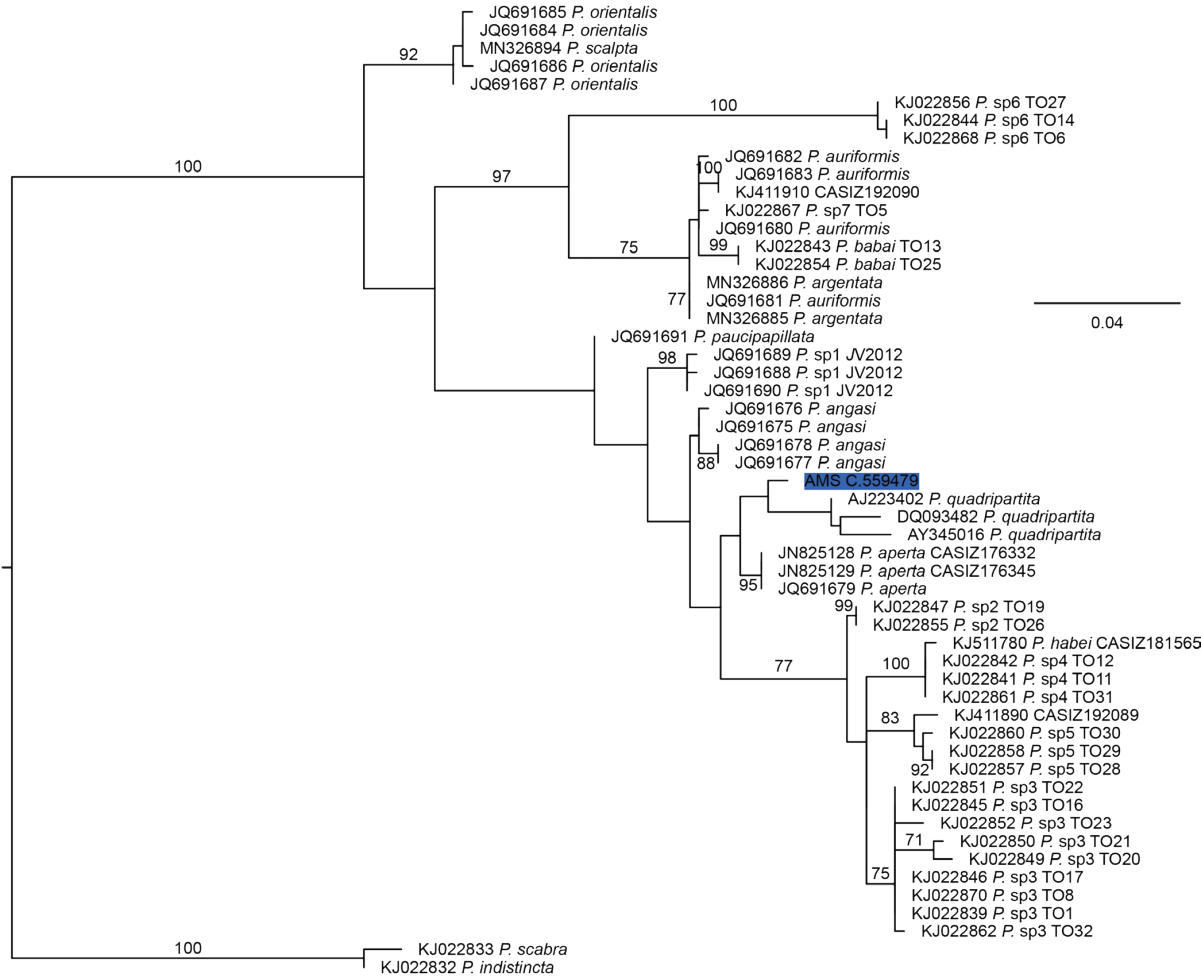
Phylogenetic relationships of C.559479 based on maximum likelihood analysis of the reduced 16S rRNA dataset. More distant outgroups have been removed and the topology rooted on *Philinescabra* + *P.indistincta*. Numbers near nodes refer to bootstrap percentages above 70%. The scale bar indicates 0.05 changes per site. Sequences are identified by accession number and species name or informal designation recorded in GenBank except those labelled *P.quadripartita* for which the species names have been changed for reasons outlined in the text. Accessions with an sp. number designation followed by a space and “TO” with a one or two digit designation refer to sequences from Oskars et al. (2015). Note that this article refers to the undescribed species in alphabetical rather than numerical order so that sp. 4 in GenBank is identified as sp. D in Oskars et al. (2015).

The 16S rRNA alignment comprised 444 positions of which 38 were variable but not parsimony-informative and 169 which were parsimony-informative. The optimal tree found in the ML analysis of the alignment had a ln likelihood of –3619.22 and there were 650 bootstrap replicates conducted under the MRE criterion. After Gblocks filtering, there were 370 positions in the alignment, of which 30 were variable but not parsimony-informative and 133 were parsimony-informative. ML analysis of these data had a ln likelihood of –2831.37 and there were 800 bootstrap replicates conducted under the MRE criterion. The optimal tree found in the ML analysis of the reduced 16S rRNA dataset (Fig. 4) had a ln likelihood of –1787.45 and 500 bootstrap replicates were conducted under the MRE criterion. In the optimal trees based on the Gblocks-filtered data and the reduced dataset, the sequence of C.559479 was resolved but without bootstrap support as the sister group of a clade of three sequences apparently, as discussed below, from *Philinequadripartita* Ascanius, 1772. The sequence of C.559479 and the three *P.quadripartita* sequences formed two separate basal lineages in a large clade that received bootstrap support of 56% in the analysis of the complete 16S rRNA alignment. The K2P distances from C.559479 to the three *P.quadripartita* specimens averaged 0.027 (minimum 0.023).

In all analyses of the 16S rRNA data, MN326885 collected from *P.argentata* by Chaban et al. (2019) and the two *P.auriformis* Suter, 1909 sequences form a clade close to other specimens from the former species. Price et al. (2011) proposed that *P.argentata* is a synonym of *P.orientalis* Adams, 1854. The type specimen of the former has shell dimensions of 6 × 5 mm (Gould 1859) which is much smaller than those of the latter species. *Philineargentata* was maintained as a distinct species by Chaban (2014) owing to differences in gizzard plate morphology, and subsequently by Chaban et al. (2019). The Chaban et al. (2019) sequence of *P.scalpta* A. Adams, 1862 (MN326894) was unexpectedly included with robust bootstrap support in a clade of sequences supposed to be from *P.orientalis* that were collected by Krug et al. (2012).

The 28S rRNA D1 alignment (Suppl. material 2: Fig. S1) comprised 337 bases, 300 of which were constant, 15 parsimony-uninformative and 22 parsimony-informative. The optimal ML topology had a ln likelihood of –832.825 based on 1000 bootstrap replicates. The sequence of C.559479 was most similar to the only available sequence of *P.aperta* (Linnaeus, 1767) (K2P distance of 0.003, with the next most similar sequence having a distance of 0.006). The C.559479 and *P.aperta* specimens were not resolved as sister groups in the ML analysis but did have this relationship in minimum evolution topologies (not shown). 28S rDNA D1 sequences were not available from *P.quadripartita*.

The histone H3 alignment (Suppl. material 3: Fig. S2) comprised 365 bases, of which 258 were constant, 23 parsimony-uninformative and 84 parsimony-informative. The optimal ML topology had a ln likelihood of –1819.273 based on 500 bootstrap replicates conducted under the MRE criterion. The sequence of C.559479 was included in a large clade (bootstrap support 81%) with sequences from *P.scalpta*, *P.japonica*, and five undescribed species (Oskars et al. 2015) from the Philippines, southwest Pacific, and Taiwan (one specimen: accession KJ022956). The latter specimen was collected from deep water (326–331 m) off northeastern Taiwan (24°48'22.8024"N, 122°07'58.206"E). The most similar sequence to C.559479 was from Panglao in the Philippines (KJ022971, K2P distance 0.012), with the next most similar (K2P distance 0.015) being from *P.aperta* (DQ093508) and the Taiwanese specimen.

## Discussion

Morphologically, C.559479 has more apparent affinity with *P.otukai* Habe, 1946, than other Taiwanese species. If the taxa were conspecific, this would represent a very large increase in the depth range of *P.otukai* which is reported to extend only to 100 metres (Lin 2004). The definite identification of C.559479 as *P.otukai* would, however, be premature because of the lack of relevant morphological information for that species. The shell of *P.otukai* is generally larger than that of C.559479, with the type specimen (Otuka 1936: figs 22, 23, as “*P.aperta*”) of the former having a length of 24 mm and width of 17 mm (Otuka 1936). Although Lee (1998) gives a minimum of 15 mm (in a range of 15–28 mm) most reports from other regions (e.g., Lin 2004) suggest that the shell is much longer (> 25 mm). The gizzard plates of C.559479 appear similar in shape to those of *P.otukai* illustrated by Lee (1998) depicting the inner surface. However, no pores are visible in the illustrations of the outer surfaces of the plates in Lee (2018).

Specimen C.559479 is readily distinguished from *P.argentata*, which has two slit-like recesses on the outer surfaces of its gizzard plates (Chaban and Chernyshev 2014), that are absent here (Fig. 3). *Philineargentata* and *P.orientalis* were synonymised by (Price et al. 2011). However, they were treated as distinct species by Chaban (2014) so the possibility that C.559479 belongs to *P.orientalis* may be considered. The specimen C.559479 is distinct externally from *P.orientalis* sensu (Price et al. 2011) in its smaller size and creamy yellow headshield colour (vs. white in the latter species). C.559479 has a large pair of circular pores on all gizzard plates, whereas the pores are small in *P.orientalis*Price et al. (2011).

Specimen C.559479 differs from *P.vitrea* in the small size of the latter (shell dimensions of the type specimen 10 × 8 × 3 mm: Gould 1859). It also differs in the apparently much lower projection of the upper end of the outer lip of *P.vitrea*, allowing for the damaged condition of this shell section in the specimen studied here.

Sufficient information is available to distinguish C.559479 morphologically from only a few other regional species of *Philine*. These include *P.scalpta* in which the gizzard plates are quasi-trapezoidal with elongated pores (Chaban and Chernyshev 2014). The shell of the shallow water *P.kinglipini* Tchang, 1934 differs by the absence of spiral sculpture (Lin 1997: 198). *Philinekinglipini* attains a larger size of ~ 40–42 mm length and 18–19 mm width with shell dimensions of 19–20 mm × 14–14.5 mm (Lin 1997). The gizzard plates of *P.kinglipini* are all dissimilar to each other in size (Tchang 1934), with the second largest being quasi-trapezoidal. Both arms on all three plates of *P.kinglipini* are broad and nearly equal in length.

*Philinepaucipapillata* Price, Gosliner & Valdés, 2011 was described as a new species from the South China Sea. Specimen C.559479 is readily distinguished morphologically from this species in coloration and size (*P.paucipapillata* is uniformly white and ~ 4–5 cm in length). The pores in the gizzard plates of *P.paucipapillata* are minute (Price et al. 2011) in contrast to those of C.559479.

*Philinekurodai* Habe, 1946, described from Japan, has been reported from Taiwan although the Taiwanese records are now regarded as being based on specimens of *P.otukai* (Lee 1998; Wu 2004). However, although both *P.otukai* and *P.kurodai* were described in Habe (1946) no characters distinguishing them from each other were listed and we have been unable to find any subsequent discussion discriminating these species. Neither *P.kurodai* nor *P.otukai* was mentioned by Price et al. (2011) but both are accepted by Chaban (2016).

Three species from northeastern Asia, *P.acutangula* Adams, 1862, *P.crenata* Adams, 1862, and *P.coreanica* Adams, 1855, have received little recent attention, particularly after Herdman (1906) made the latter two synonyms of “*Philineaperta*” (Table 1). The species all appear to belong to the group of species described by Adams (1855, 1862) categorised by Kurodai and Habe (1954) as “unobvious” and are presently best regarded as species inquirenda. To the best of our knowledge, the locations of their type specimens are unknown.

Analyses including the DNA sequences of C.559479 collected here highlight the taxonomic complexity of *Philine* in northeastern Asia. The available 16S rRNA data from the region represent at least five species. One of these species is represented by the C.559479 sequence from Taiwan. The other species are *P.paucipapillata*, *P.argentata* sensu Chaban et al. (2019), a species uncertainly referred to “*P.orientalis*”, *P.japonica* or *P.scalpta* – see below), and an additional species, denoted “species D” by Oskars et al. (2015), which is represented by histone H3 DNA sequences. The latter may have affinity with *Philinehabei* Valdés, 2008. The Taiwanese specimen of “species D” lacks 16S rDNA sequences but the other specimens referred to it by Oskars et al. (2015) are found in analyses of this gene (Fig. 4) in a strongly supported clade with the *P.habei* sequence (KJ511780). This clade clearly excludes C.559479. The gizzard plates of *P.habei* have large elongate pores (Valdés 2008; Price et al. 2011) that appear very different from those of C.559479.

The three sequences of 16S rRNA that were most similar to C.559479 are recorded in GenBank as belonging to *Philineaperta*. However, the true identity of the specimens from which these sequences were derived appears to be *P.quadripartita*, a species from the northern Atlantic Ocean and Mediterranean Sea. The sequences form a distinct lineage separate from the two South African specimens from near the type locality of true *P.aperta*, and were determined from specimens taken within the range of *P.quadripartita*: AY345016 derives from a specimen from south-eastern Spain (Grande et al. 2004); and both AJ223402 (Thollesen 1999) and DQ093482 (Aktipis et al. 2008) are from Sweden.

The “*P.orientalis*” 16S rRNA sequence accession JQ691684) from Japan (Krug et al. (2012) is from a specimen identified as *P.japonica* in the catalogue of the Natural History Museum, London (BMNH:1996409). This is included in a clade with three Californian (non-native range) haplotypes of “*P.orientalis*” sequenced by Krug et al. (2012) and another from a specimen identified by Chaban et al. (2019) as *P.scalpta*, a species that was redescribed by Chaban and Chernyshev (2014) who removed it from the synonymy of *P.vitrea* in which it had been placed by Higo et al. (1999). If *P.japonica* and *P.scalpta* are, as maintained by Chaban et al. (2019), distinct from *P.orientalis*, then sequences truly from the latter species are probably yet to be determined. It is possible that the sequences from the taxon from near Darwin in northern Australia labelled sp. 1 JV 2012 in GenBank (Krug et al. 2012) may belong to *P.orientalis* or a closely related species. The named species to which these sequences are most similar are the Australasian *Philineangasi* (Crosse, 1865, in Crosse and Fischer 1865) and *P.paucipapillata*. Some radular and penial morphology characters distinguish *P.paucipapillata* and *P.orientalis* but they are closely related in the morphologically based phylogeny of Price et al. (2011). In this phylogeny, *P.paucipapillata* is shown as the sister group of the pair composed of *P.orientalis* and *P.angasi*.

## Conclusions

The difficulty of identifying the unusual deep water *Philine* specimen studied here emphasises that destructive sampling should be minimised where the taxonomy is un-integrated. We tentatively suggest from examination of the external morphology and micro-CT scanning that it may have affinities with the shallow water *P.otukai* but any certainty is precluded by the lack of a description of that species, especially one detailing the range of variation it might encompass in characters such as the size of the gizzard plate pores. There is no diagnosis of *P.otukai* that would guide specimen dissection for relevant anatomical characters. Considerable further research efforts are required to provide a framework in which such characters could be sought. These include DNA studies of a wider range of regional species of *Philine*, particularly sequences definitely from *P.orientalis*, *P.kurodai*, *P.otukai*, and *P.vitrea*. Detailed morphological investigations are needed for *P.japonica*, *P.otukai*, *P.kurodai*, and *P.vitrea*. The status of species such as *P.coreanica* may remain unknown but it may possibly be resolved if shell fragments from type material ever become available for macromolecular analysis, especially proteomics.

## Supplementary materials

Supplementary material 1 Reconstructed micro-CT scan of C.559479 Data type Mpg file Explanation note Reconstructed micro-CT scan of C.559479. https://binary.pensoft.net/file/589863This dataset is made available under the Open Database License (http://opendatacommons.org/licenses/odbl/1.0/). The Open Database License (ODbL) is a license agreement intended to allow users to freely share, modify, and use this Dataset while maintaining this same freedom for others, provided that the original source and author(s) are credited.Donald J. Colgan, Shane T. Ahyong, Karine Mardon, Ian M. Brereton

Supplementary material 2 Figure S1. Image from the reconstructed micro-CT scan of C.559479 Data type Pdf file Explanation note Image from the reconstructed micro-CT scan of C.559479. Dorsal view of the shell of C.559479, with boxed area illustrating irregularly cancellate sculpture. https://binary.pensoft.net/file/589864This dataset is made available under the Open Database License (http://opendatacommons.org/licenses/odbl/1.0/). The Open Database License (ODbL) is a license agreement intended to allow users to freely share, modify, and use this Dataset while maintaining this same freedom for others, provided that the original source and author(s) are credited.Donald J. Colgan, Shane T. Ahyong, Karine Mardon, Ian M. Brereton

Supplementary material 3 Figure S2. Phylogenetic relationships of C.559479 based on maximum likelihood analysis of the D1 28S rRNA dataset Data type Pdf file Explanation note Phylogenetic relationships of C.559479 based on maximum likelihood analysis of the D1 28S rRNA dataset. Numbers near nodes refer to bootstrap percentages above 70%. Scale bar indicates 0.01 changes per site. Sequences are identified by accession number and species name or informal designation recorded in GenBank. Accessions with an sp. number designation followed by a space and “TO” with a one or two digit designation refer to sequences from Oskars et al. (2015). Note that that article refers to the undescribed species in alphabetical rather than numerical order so that sp. 4 in GenBank is identified as sp. D in Oskars et al. (2015). https://binary.pensoft.net/file/589865This dataset is made available under the Open Database License (http://opendatacommons.org/licenses/odbl/1.0/). The Open Database License (ODbL) is a license agreement intended to allow users to freely share, modify, and use this Dataset while maintaining this same freedom for others, provided that the original source and author(s) are credited.Donald J. Colgan, Shane T. Ahyong, Karine Mardon, Ian M. Brereton

Supplementary material 4 Figure S3. Phylogenetic relationships of C.559479 based on Maximum Likelihood analysis of the histone H3 dataset. Data type Pdf file Explanation note Phylogenetic relationships of C.559479 based on Maximum Likelihood analysis of the histone H3 dataset. Numbers near nodes refer to bootstrap percentages above 70%. The scale bar indicates 0.05 changes per site. Sequences are identified by accession number and species name or informal designation recorded in GenBank. Accessions with a sp. number designation followed by a space and “TO” with a one or two digit designation refer to sequences from Oskars et al. (2015). Note that this article refers to the undescribed species in alphabetical rather than numerical order so that sp. 4 in GenBank is identified as sp. D in Oskars et al. (2015). The sequence indicated by a red square is the Taiwanese specimen with apparent affinity to P.habei (see text). https://binary.pensoft.net/file/589866This dataset is made available under the Open Database License (http://opendatacommons.org/licenses/odbl/1.0/). The Open Database License (ODbL) is a license agreement intended to allow users to freely share, modify, and use this Dataset while maintaining this same freedom for others, provided that the original source and author(s) are credited.Donald J. Colgan, Shane T. Ahyong, Karine Mardon, Ian M. Brereton

## References

[B1] Adams A (1854) Descriptions of some new species of Lophocercidae and Philinidae, from the Cumingian Collection. Proceedings of the Zoological Society of London 22: 94–95. 10.1111/j.1469-7998.1854.tb07238.x

[B2] Adams A (1862) On some new species of Cylichnidae, Bullidae and Philinidae. Annals and Magazine of Natural History, Series 3, 9: 150–161. 10.1080/00222936208681198

[B3] Aktipis SW Giribet G Lindberg DR Ponder WF (2008) Gastropoda: an overview and analysis. In: Ponder WF Lindberg DR (Eds) Phylogeny and Evolution of the Mollusca.University of California Press, Berkeley, 201–237. 10.1525/california/9780520250925.003.0009

[B4] Castresana J (2000) Selection of conserved blocks from multiple alignments for their use in phylogenetic analysis. Molecular Biology and Evolution 17: 540–552. 10.1093/oxfordjournals.molbev.a026334 10742046

[B5] Chaban EM Chernyshev AV (2014) Opisthobranch cephalaspidean mollusks (Gastropoda: Opisthobranchia) of Vostok Bay, Sea of Japan. Part 1. The Bulletin of the Russian Far East Malacological Society 18: 41–62. [In Russian]

[B6] Chaban EM (2014) Notes on *Yokoyamaiaornatissima* (Yokoyama, 1927) and *Philineargentata* Gould, 1859 (Opisthobranchia: Cephalaspidea: Philinidae) from the Sea of Japan. Abstracts of the Conference Mollusks of the Eastern Asia and Adjacent Seas October 6–8, 2014, Vladivostok, Russia, 7–10.

[B7] Chaban EM (2016) Heterobranch mollusks of the orders Acteonoidea and Cephalaspidea (Gastropoda: Heterobranchia) of Vietnam: annotated check-list with illustrations of some species. In: Adrianov AV Lutaenko KA (Eds) Biodiversity of the western part of the South China Sea.Dalnauka, Vladivostok, 415–448.

[B8] Chaban EM Ekimova IA Schepetov DM Kohnert PC Schroedl M Chernyshev AV (2019) Euopisthobranch mollusks of the order Cephalaspidea (Gastropoda: Heterobranchia) of the Kuril-Kamchatka Trench and the adjacent Pacific abyssal plain with descriptions of three new species of the genus *Spiraphiline* (Philinidae). Progress in Oceanography 178: e102185. 10.1016/j.pocean.2019.102185

[B9] Colgan DJ Da Costa P (2009) DNA haplotypes cross species and biogeographic boundaries in estuarine hydrobiid snails of the genus *Tatea*. Marine and Freshwater Research 60: 861–872. 10.1071/MF08200

[B10] Colgan DJ Ponder WF Eggler PE (2000) Gastropod evolutionary rates and phylogenetic relationships assessed using partial 28S rDNA and histone H3 sequences. Zoologica Scripta 29: 29–63. 10.1046/j.1463-6409.2000.00021.x

[B11] Colgan DJ Ponder WF Beacham E Macaranas JM (2003) Molecular phylogenetic studies of Gastropoda based on six gene segments representing coding or non-coding and mitochondrial or nuclear DNA. Molluscan Research 23: 123–148. 10.1071/MR03002

[B12] Crosse H Fischer P (1865) Description d’espèces nouvelles de l’Australie méridionale. Journal de Conchyliologie 13: 38–55.

[B13] Gonzales C Gosliner T (2014) Six new species of *Philine* (Opisthobranchia: Philinidae) from the tropical Indo-Pacific. In: Williams GC Gosliner TM (Eds) The Coral Triangle: The 2011 Hearst Philippine Biodiversity Expedition.California Academy of Sciences, San Francisco, 351–383. 10.5962/bhl.title.154474

[B14] Grande C Templado J Cervera JL Zardoya R (2004) Phylogenetic relationships among Opisthobranchia (Mollusca: Gastropoda) based on mitochondrial *cox 1*, *trnV*, and *rrnL* genes. Molecular Phylogenetics and Evolution 33: 378–388. 10.1016/j.ympev.2004.06.008-388 15336672

[B15] Habe T (1946) Report of the CephalaspideaOpisthobranchia in Japan. Venus 14: 183–190. [In Japanese]

[B16] Habe T (1950) Philinidae in Japan. In: Kuroda T (Ed.) Illustrated catalogue of Japanese shells 8: 48–52. Kyoto, Kairui Bunken Kankokai.

[B17] Habe T (1954) Report on the Mollusca chiefly collected by the S. S. Soyo-Maru of the Imperial Fisheries Experimental Station of the continental shelf bordering Japan during the years 1922–1930. Part 1. Cephalaspidea. Publications of the Seto Marine Biological Laboratory 3: 301–318. [pl. 38] 10.5134/174489

[B18] Hamatani I (1961) Notes on veligers of Japanese opisthobranchs (3). Publications of the Seto Marine Biological Laboratory 9: 67–79. 10.5134/174661

[B19] Herdman WA (1906) Report to the government of Ceylon on the pearl oyster fisheries of the Gulf of Manaar. The Royal Society, London, 384 pp. [p. 348]

[B20] Higo SI Callomon P Gotō Y (1999) Catalogue and bibliography of the marine shell bearing Mollusca of Japan: Gastropoda, Bivalvia, Polyplacophora, Scaphopoda. Elle Scientific Publications, Osaka, 749 pp.

[B21] Krug PJ Asif JH Baeza I Morley MS Blom WM Gosliner TM (2012) Molecular identification of two species of the carnivorous sea slug *Philine*, invaders of the US west coast. Biological Invasions 14: 2447–2459. 10.1007/s10530-012-0242-9

[B22] Kumar S Stecher G Tamura K (2016) MEGA7: Molecular Evolutionary Genetics Analysis version 70 for bigger datasets. Molecular Biology and Evolution 33: 1870–1874. 10.1093/molbev/msw054 27004904 PMC8210823

[B23] Kuroda T Habe T (1954) On some Japanese Mollusca described by A. Adams, whose specimens are deposited in the Redpath Museum of Canada (No. 1). Venus 18: 1–16. [pls 1, 2]

[B24] Lee C-T (2018) *Philineotukai* 25.50mm. http://leechitse66.blogspot.com.au/2012/10/philine-otukai-2550mm.html [Accessed 6.II.2018]

[B25] Lee YC (1998) Thin bubble shells in Taiwan. The Pei-Yo 24: 13–15. [In Chinese]

[B26] Lee YC Wu WL (2005) 作伙去撿螺仔-台灣常見貝類彩色圖誌 [The Taiwan common mollusks in colour]. Forestry Bureau, Council of Agriculture, Taipei, 294 pp. [In Chinese]

[B27] Lin G (1997) Fauna Sinica, Phylum Mollusca, Class Gastropoda, Subclass Opisthobranchia, Order Cephalaspidea. Science Press, Beijing, 246 pp. [28 pls.] [In Chinese with English summary]

[B28] Lin G (2004) Opisthobranchia. In: Qi Z (Chief Ed.) Seashells of China. China Ocean Press, Beijing, 224–234.

[B29] Lischke CE (1872) Diagnosen neuer Meeres-Conchylien von Japan. Malakozoologische Blätter 19: 100–109. [In German]

[B30] McArthur AG Koop BF (1999) Partial 28S rDNA sequences and the antiquity of the hydrothermal vent endemic gastropods. Molecular Phylogenetics and Evolution 13: 255–274. 10.1006/mpev.1999.0645 10603255

[B31] Miller MA Pfeiffer W Schwartz T (2010) Creating the CIPRES Science Gateway for inference of large phylogenetic trees. In IEEE (consortium), Proceedings of the Gateway Computing Environments Workshop (GCE), 14 Nov 2010, New Orleans, 1–8. 10.1109/GCE.2010.5676129

[B32] Morton B Chiu ST (1990) The diet, prey size and consumption of *Philineorientalis* (Opisthobranchia: Philinidae) in Hong Kong. Journal of Molluscan Studies 56: 289–299. 10.1093/mollus/56.2.289

[B33] Ohnheiser LT Malaquias M (2013) Systematic revision of the gastropod family Philinidae (Mollusca: Cephalaspidea) in the north-east Atlantic Ocean with emphasis on the Scandinavian Peninsula. Zoological Journal of the Linnean Society 167: 273–326. 10.1111/zoj.12000

[B34] Oskars TR Bouchet P Malaquias MAE (2015) A new phylogeny of the Cephalaspidea (Gastropoda: Heterobranchia) based on expanded taxon sampling and gene markers. Molecular Phylogenetics and Evolution 89: 130–150. 10.1016/j.ympev.2015.04.011 25916189

[B35] Otuka Y (1936) Notes on some shells from southern Taiwan. Venus 6: 155–162. [in Japanese]

[B36] Palumbi SR (1996) Nucleic acids II: the polymerase chain reaction. In: Hillis DM Moritz C Mable BK (Eds) Molecular Systematics.Sinauer & Associates, Sunderland, Massachusetts, 205–247.

[B37] Pattengale ND Alipour M Bininda-Emonds ORP Moret BME Stamatakis A (2010) How many bootstrap replicates are necessary? Journal of Computational Biology 17: 337–354. 10.1007/978-3-642-02008-7_13 20377449

[B38] Pilsbry HA (1895) Philinidae. In: Tryon GW Pilsbry HA (Eds) Manual of conchology; structural and systematic with illustrations of the species.Academy of Natural Sciences of Philadelphia 16: 2–28.

[B39] Price RM Gosliner TM Valdés Á (2011) Systematics and phylogeny of *Philine* (Gastropoda: Opisthobranchia), with emphasis on the *Philineaperta* species complex. The Veliger 51: 1–58.

[B40] Shepelenko M Brumfeld V Cohen SR Klein E Lubinevsky H Addadi L Weiner S (2015) The gizzard plates in the cephalaspidean gastropod *Philinequadripartita*: Analysis of structure and function. Quaternary International 390: 4–14. 10.1016/j.quaint.2015.04.060

[B41] Sowerby GB (1855) Thesaurus conchyliorum. Monographs of genera of shells. Sowerby, London, 899 pp. [Vol. 2, p. 601, pl. 125]

[B42] Stamatakis A Hoover P Rougemont J (2008) A rapid bootstrap algorithm for the RAxML web-servers. Systematic Biology 75: 758–771. 10.1080/10635150802429642 18853362

[B43] Tanaka Y. 1958. Studies on molluscan larvae (I). Venus 20: 207–219.

[B44] Tapparone-Canefri C (1874) Malacologia (Gasteropodi, Acefali e Brachiopodi). Zoologia del Viaggio Intorno al Globo della regia fregata Magenta durante gli anni 1865–68. Stamperia reale di G.B. Paravia e Comp, Torino, 162 pp. [4 pls.] [Explanation and figure, pl. 2] [In Italian] 10.5962/bhl.title.11193

[B45] Taylor JD (1982) Diets of sublittoral predatory gastropods of Hong Kong. In: Morton B Tseng CK (Eds) Proceedings of the First International Marine Biological Workshop.Hong Kong University Press, Hong Kong, 907–920.

[B46] Tchang S (1934) Contribution à l’étude de Opistobranchea de la Côte de Tsingtao. Contributions from the Institute of Zoology, National Academy of Peiping 2(2): 1–148. [pls 1–16] [In French]

[B47] Thollesson M (1999) Phylogenetic analysis of Euthyneura (Gastropoda) by means of the 16S rRNA gene: use of a ‘fast’gene for ‘higher-level’ phylogenies. Proceedings of the Royal Society of London B: Biological Sciences 266: 75–83. 10.1098/rspb.1999.0606

[B48] Thompson JD Gibson TJ Plewniak F Jeanmougin F Higgins DG (1997) The CLUSTAL X windows interface: flexible strategies for multiple sequence alignment aided by quality analysis tools. Nucleic Acids Research 25: 4876–4882. 10.1093/nar/25.24.4876 9396791 PMC147148

[B49] Valdés AA (2008) Deep-sea” cephalaspidean” heterobranchs (Gastropoda) from the tropical southwest Pacific. In: Héros V Cowie RH Bouchet P (Eds) Tropical Deep-Sea Benthos 25.Mémoires du Muséum national d’Histoire naturelle 196: 587–792.

[B50] Wu W-L (2004) List of Taiwan Malacofauna. IV, Gastropoda, Heterogastropoda, Heteropoda, Opisthobranchia and Pulmonata. The Council of Agriculture, Taipei, 160 pp.

[B51] Zhang S. (2008) Order Cephalaspidea. In: Liu JY (Ed.) Checklist of marine biota of China seas.Science Press, Academia Sinica, Beijing, 527–532.

