# Supplementary figures and images for: Rare specimen identification in an un-integrated taxonomy: implications of DNA sequences from a Taiwanese Philine (Mollusca, Philinidae)
Source: Zookeys. 2021 Sep 17;1060:93–110. doi: 10.3897/zookeys.1060.28809 (PMC8463522; doi:10.3897/zookeys.1060.28809)

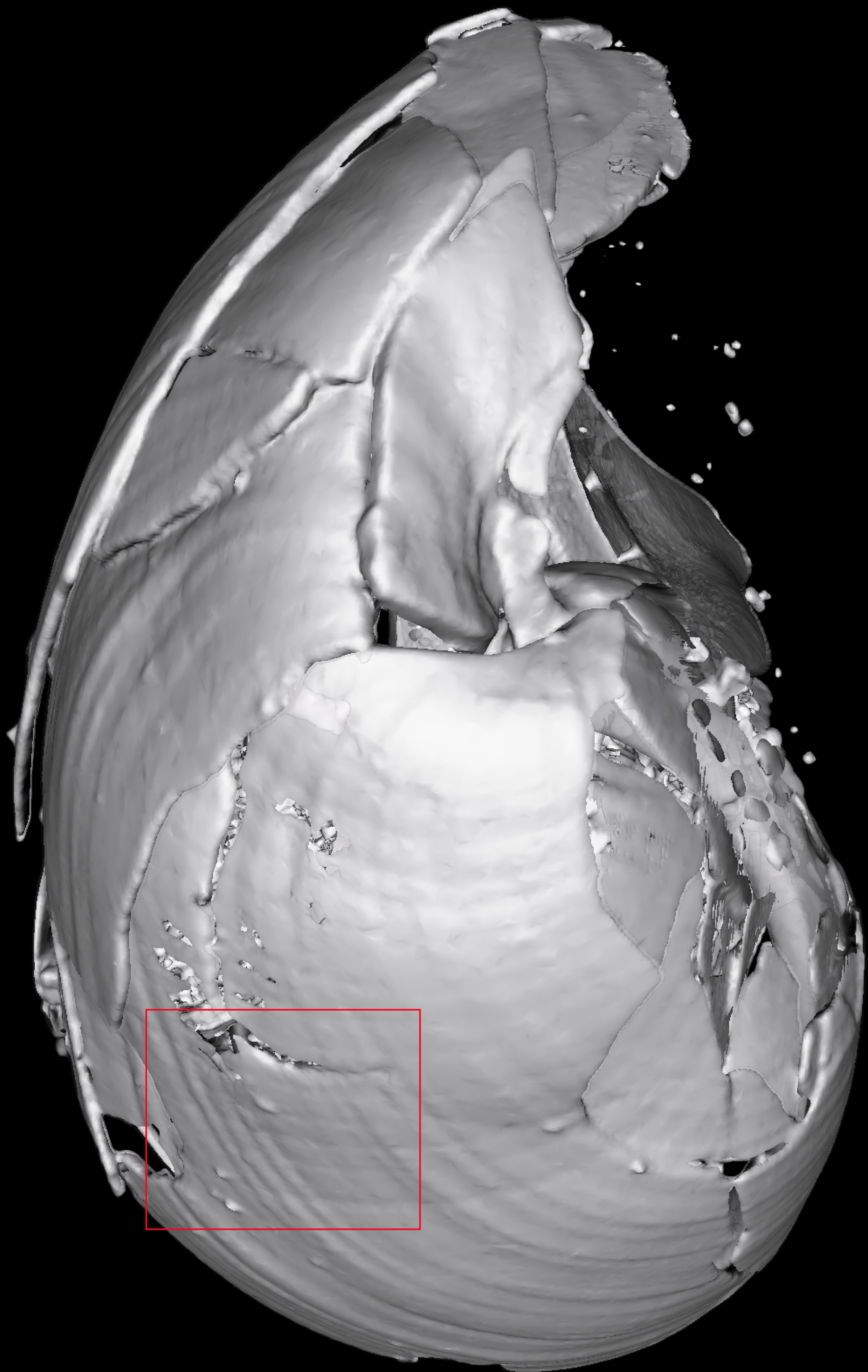

Supplement: Supplementary material 2 — Figure S1. Image from the reconstructed micro-CT scan of C.559479 [file zookeys-1060-093-s002.pdf]

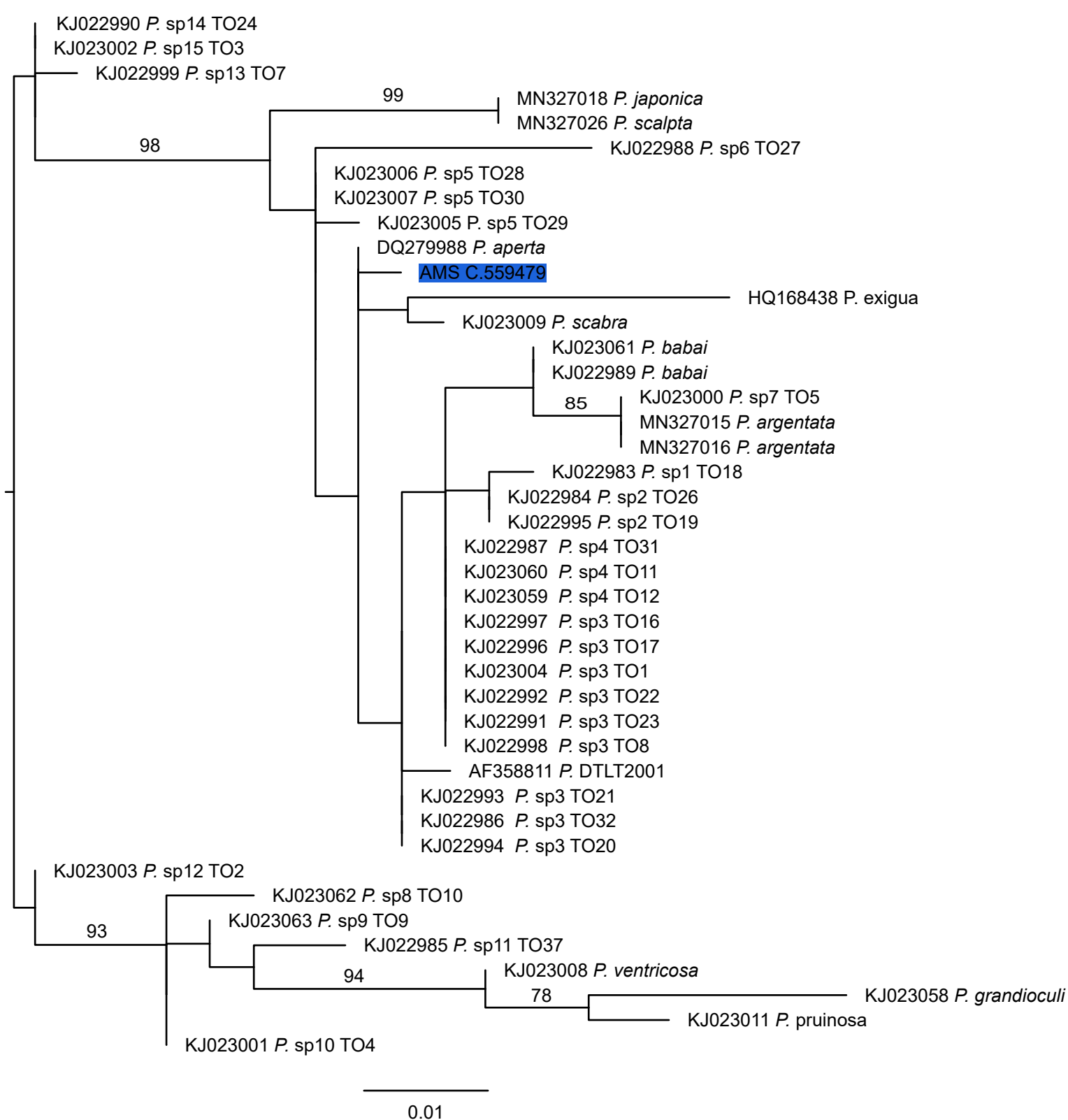

Supplement: Supplementary material 3 — Figure S2. Phylogenetic relationships of C.559479 based on maximum likelihood analysis of the D1 28S rRNA dataset [file zookeys-1060-093-s003.pdf]

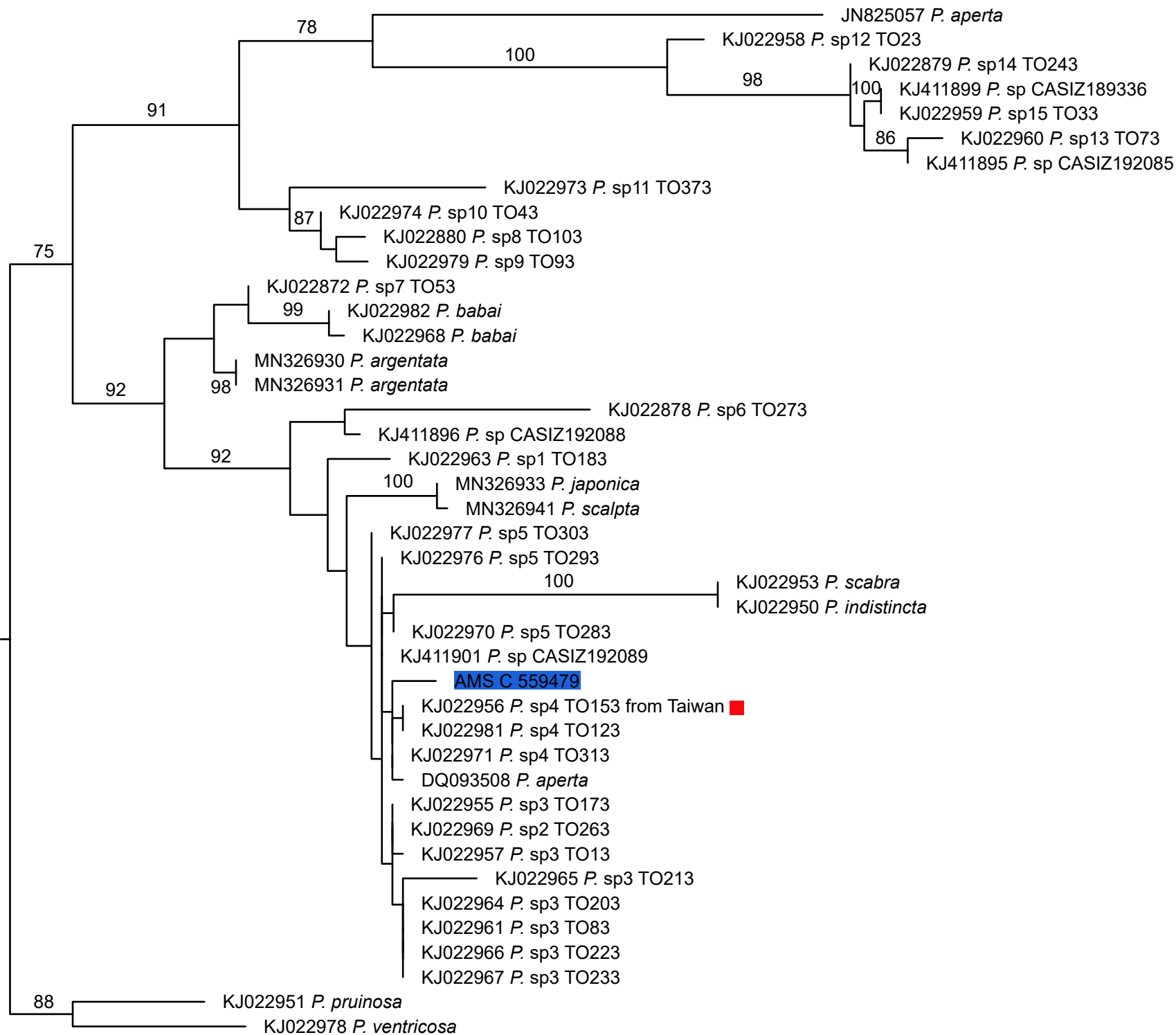

Supplement: Supplementary material 4 — Figure S3. Phylogenetic relationships of C.559479 based on Maximum Likelihood analysis of the histone H3 dataset. [file zookeys-1060-093-s004.pdf]
